# Hemispheric Specialization of the Primate Inferior Parietal Lobule

**DOI:** 10.1007/s12264-021-00807-4

**Published:** 2021-12-29

**Authors:** Sam Vickery, Simon B Eickhoff, Patrick Friedrich

**Affiliations:** 1 Institute of Neuroscience and Medicine, Brain & Behaviour (INM-7), Research Centre Jülich, 52428 Jülich, Germany; 2 Institute of Systems Neuroscience, Medical Faculty, Heinrich-Heine University Düsseldorf, 40225 Düsseldorf, Germany

Hemispheric asymmetries can be seen as one of the evolutionary adaptations that allowed the human brain to muster more complex cognitive processes than other primates. In this vein, the study published by Cheng *et al*. [1] presents a pivotal investigation of both the regional and connectional asymmetries within the inferior parietal lobule (IPL) in human, chimpanzee, and macaque. By investigating 4 sub-divisions of the IPL across the three species, Cheng and colleagues showed that the macroanatomical and connectional architecture of the IPL became more asymmetric throughout the primate lineage. While macaques show little to no structural asymmetries, chimpanzees display a more asymmetric architecture but with both leftward and rightward asymmetries in various connections. In contrast, the human IPL displayed the highest number of asymmetries among the three species with a clear tendency towards more lateralization. This evolutionary trend towards a more lateralized organization of the IPL may have accompanied an improved command of tool-use, stronger forelimb asymmetries, and the increasing complexity of communicative behavior.

The IPL is a part of the primate association cortex that plays an important role in language and tool-use along with a multitude of different functions [2]. Given its functional diversity and heterogeneity, a comprehensive analysis of the IPL’s macroanatomy and connectivity requires investigation on a sub-division level. However, translating a set of sub-regions from one species to another poses a central challenge for comparative research. There are various strategies to solve the issue of inter-species comparison, such as establishing a common feature space between species based on structure and/or functional measures [3], or one can investigate homologous regions and/or features in each species. To accomplish this task, Cheng et al. implemented the latter by using a connectivity-based parcellation approach, which creates sub-divisions based on diffusion-weighted probabilistic tractography. This approach can give several solutions that vary in the number of sub-divisions. The authors choose a 4-cluster solution with divisions that follow a rostral-to-caudal (anterior-posterior) organization in macaques, chimpanzees, and humans. This solution maximizes the similarity across species and is consistent with previously reported anatomical and cytoarchitecture parcellations.

**Fig. 1:**
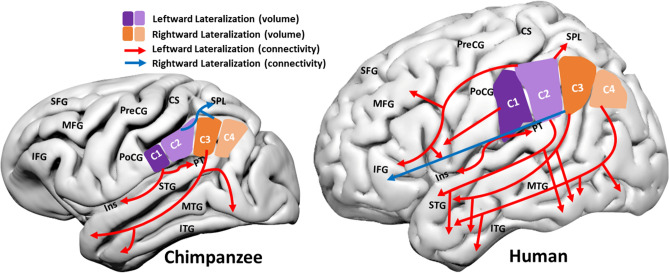
An adaptation of the graphical abstract originally provided in Cheng *et al*. which represents the connectivity and volumetric asymmetries found in chimpanzees and humans. The results are presented on a surface projection of the chimpanzee reference template (JunaChimp [4]) and human (MNI). C1:C4, subdivisions of the IPL; CS, central sulcus; IFG, inferior frontal gyrus; Ins, insula; ITG, inferior temporal gyrus; MFG, middle frontal gyrus; MTG, middle temporal gyrus; PoCG, postcentral gyrus; PreCG. precentral gyrus; PT, planum temporale; SFG, superior frontal gyrus; SPL, superior parietal lobule.

Utilizing the IPL subdivisions derived by connectivity-based parcellation, Cheng *et al.* conducted a thorough exploration of the structural asymmetries within the IPL across the three species. The investigation was centered around the gray matter (GM) volume, probabilistic white matter (WM) connections, and the cortical surface vertices of the WM connections. Regarding the different structural measures, the old-world monkeys (macaques) did not present any asymmetries while the great apes (chimpanzees and humans) showed similar and divergent asymmetrical organization. In great apes, rostral subdivisions were leftward asymmetric and caudal subdivisions were rightward asymmetric. This indicates a switch from a symmetrical to an asymmetrical organization in the IPL in a common ancestor of the great apes and old-world monkeys. Asymmetric WM connection from the IPL sub-divisions and the resulting cortical surface regions of said connections is where Cheng and colleagues found divergences from chimpanzees to humans. Humans presented more plentiful lateralized IPL connections, in particular those towards the left hemisphere, as well as to unique cortical areas such as the ventral frontal cortex, motor cortex, and the lateral temporal cortex as compared to chimpanzees (Fig. 1). Cheng and colleagues propose that this increase in organizational asymmetries may have contributed to the evolution of language, tool-use, and handedness in the primate lineage.

The leftward asymmetry of connectivity between the IPL and primary motor cortex (M1) appears particularly interesting, given its potential implications for manual skills as outlined by the authors. Human handedness is an unprecedented example of behavioral laterality, as it is strongly skewed towards a population-wide and task-invariant preference of the right hand, which is unparalleled in other vertebrates [5]. Comparisons of primate handedness indicate a potential evolutionary continuum of manual dexterity, given that evidence points towards a population-level handedness in chimpanzees, which is however less robustly expressed than human handedness [6]. The leftward asymmetric IPL–M1 connectivity might represent an important evolutionary adaptation that contributed to the increased left-hemispheric dominance in motor functions from chimpanzee to human. As such, this finding aligns with the idea that the sensory-motor system may have evolved to form the foundation of increased manual skills [7], including tool-use.

While this work makes an important contribution to our understanding of the potential role of connectional brain asymmetries in primate brain evolution, it also gives some important leads for future studies. For instance, one topic concerns the relationship between structure and function. It is known that brain regions can be functionally connected and thus take part in the same functional network, albeit in the absence of direct structural connections [8]. Structure and function are especially decoupled in association cortices compared to primary sensory and motor cortices [9]. Therefore, the effect of IPL connections and their asymmetry on functional networks and behavior need further investigation.

In a similar vein, Cheng and colleagues show group-level differences in IPL asymmetries between the three species. The variability and individual differences of these IPL asymmetries within a species grant another perspective that may help to unravel their functional relevance. A study by Croxson and colleagues [10] indicates that WM is more variable than GM in humans and macaque monkeys. However, the human brain is generally more variable than the macaque monkey’s brain, implying a higher degree of individual differences in the human brain’s architecture. Although the main findings of Cheng *et al*. did not address individual differences, such difference in IPL asymmetries is indeed important and demands further study to link to individual performance in the functional domains.

In summary, the observations made by Cheng *et al*. advance our understanding of the presence and evolution of anatomical and connectional asymmetries. In doing so, this study marks an important step towards a better understanding of how evolutionary changes in structural asymmetries may have contributed to the evolution of primate cognition.
